# Visual loss after aesthetic facial filler injection: a literature review on an ophthalmologic issue

**DOI:** 10.5935/0004-2749.20220048

**Published:** 2022

**Authors:** Juliana Mika Kato, Suzana Matayoshi

**Affiliations:** 1 Department of Ophthalmology, Hospital das Clínicas, Faculdade de Medicina, Universidade de São Paulo, Sao Paulo, SP, Brazil

**Keywords:** Dermal filler, Injection, Cosmetic technique/adverse effect, Retinal artery occlusion, Vision, low/etiology, Preenchedor dérmico, Injeção, Técnica cosmética/ efeito adverso, Oclusão da artéria retiniana, Baixa visão/etiologia

## Abstract

Dermal filler injection is among facial rejuvenation treatments that have been increasingly used. Despite being a minimally invasive procedure, it can lead to severe complications such as blindness. A review of all cases of filler-induced visual loss in the world literature was conducted to summarize the mechanisms, anatomical considerations, and clinical ophthalmologic course, current strategies of prevention and management, and trends over the years. We identified 233 cases of filler-induced visual loss, and 172 patients had a severe visual impairment in at least one eye. The typical patients are young women who received injections of hyaluronic acid or autologous fat in the glabella or nose, and the typical presentations were sudden ocular pain, ptosis, and ophthalmoplegia due to vascular occlusion. The findings of this study also suggest an increase in the number of unlicensed professionals performing the procedure. Even though the continued development of dermal fillers has improved the treatment options available, further studies and strategies are necessary to reduce the incidence and minimize the consequences of filler-induced visual loss.

## Abstract (pt)

O uso de preenchedores dérmicos é uma prática bem estabelecida de rejuvenescimento facial. Embora seja um procedimento minimamente invasivo, pode levar a complicações graves como cegueira. Uma revisão de casos de perda visual pós preenchimento facial estético foi conduzida para descrever os mecanismos, considerações anatômicas, quadro oftalmológico, atuais estratégias de prevenção e manejo, e tendências ao longo dos anos. Foram identificados 233 casos, e 172 pacientes tiveram ao menos um olho com baixa visão ao final do seguimento. O paciente típico é uma mulher jovem submetida a preenchimento de ácido hialurônico na glabela ou nariz, apresentando dor ocular súbita, ptose e oftalmoplegia devido à oclusão vascular. Este estudo também destaca um possível aumento de profissionais não habilitados realizando este procedimento. Apesar do contínuo desenvolvimento dos preenchedores dérmicos e aprimoramento das opções de tratamento disponíveis, mais estudos e estratégias são necessários para reduzir a incidência de complicações e minimizar suas consequências.

## INTRODUCTION

As the life expectancy is increasing, more people now seek procedures to counterbalance facial aging. Dermal fillers are gel-like substances injected under the skin to increase volume through a fast and low-cost procedure with minimum pain. According to the International Society of Aesthetic Plastic Surgery, >10 million injectable were used in 2018 alone worldwide, and this number is expected to increase in the next years^(1)^. Although the risk of complications is low, dermal fillers can be disastrous, as they can cause blindness, stroke, or even death when injected in the face.

This minimally invasive procedure is usually performed by dermatologists and plastic surgeons. However, complications that lead to visual loss require immediate referral to an ophthalmologist. A review of literature was conducted to summarize the mechanisms, vascular anatomic considerations, clinical course, and current strategies of prevention and management of filler-induced visual loss to facilitate eye care. To date, this is the largest review of case reports regarding blindness caused by fillers that is not limited to the English language. A comparison between the period 2015-2020 and an earlier period was performed to evaluate the changing trends.

## METHODS

**Figure 1:**
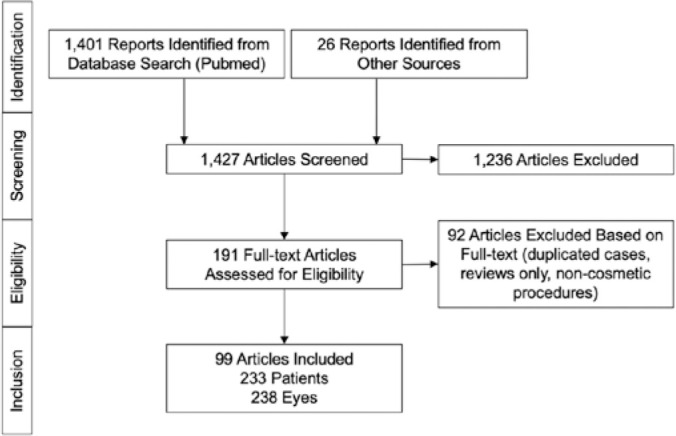
Flow diagram of the literature review process for case reports of filler-induced visual loss.

A literature search was performed to identify case reports related to visual loss after facial filler injection. The following keywords were used in the literature search on PubMed: (filler OR dermal filler OR soft tissue OR autologous fat OR hyaluronic acid OR calcium hydroxyapatite) AND (blindness OR visual loss OR vision loss OR ocular complication OR ophthalmologic complication OR retinal artery occlusion OR ophthalmic occlusion OR retina). Additional references identified from the bibliographies of pertinent articles were also included. Owing to the low incidence of blindness after filler injection, an intentionally broad search strategy was developed to identify all case reports. A flow diagram of the study is provided in figure 1. No limits were set on language to allow inclusion of more cases for analyzing this rare complication. Studies that used other substances such as corticosteroids for non-cosmetic reasons were excluded.

The data obtained from the case reports were the year of publication, country of study, age, sex, past history, laterality of the affected eye, person who performed the injection, filler type, injection site, injection instrument used, initial signs and symptoms, eye examination performed (including slit-lamp examination, tonometry, fundoscopy, fluorescein angiography, optical coherence tomography, and visual field test), brain imaging, best-corrected visual acuity (BCVA) at the initial and final presentations, time to hospital admission, treatment, follow-up duration, and sequelae.

Each case was classified according to the primary diagnosis that resulted in visual loss. If applicable, the site of vascular occlusion was classified as follows: 1) ophthalmic artery occlusion (OAO), 2) generalized posterior ciliary artery occlusion (PCAO), 3) central retinal artery occlusion (CRAO), 4) localized PCAO, and 5) branch retinal artery occlusion (BRAO).

The visual acuity measurements were converted to the logMAR scale. Counting fingers, hand motion, light perception, and no light perception (NLP) were converted to 1.9, 2.3, 2.7, and 3.0, respectively. A comparative analysis was performed between autologous fat and hyaluronic acid, and between two different periods. Statistical analysis was performed using the IBM SPSS Statistics program for Mac OS. Categorical variables were compared using the chi-square test or Fisher exact test. Continuous variables were compared using the Mann-Whitney *U* test. P values <0.05 were considered statistically significant.

## RESULTS

Up to March 2020, 233 cases (238 eyes; 99 articles) of filler-related visual loss were reported^(2-100)^. Most studies were conducted with Asians (n=190, 81.5%) and published in the last 8 years (n=199, 85.4%). China (n=83, 35.6%) and South Korea (n=81, 34.8%) had the largest number of publications, followed by the United States (n=22, 9.4%), Taiwan (n=12, 5.2%), Thailand (n=7, 3.0%), Japan (n=4, 1.7%), and other countries (n=21, 9.0%). The first reported case was published in 1988^(2)^, and since then, the number of published cases has been increasing.

Most patients were female (211/226, 93.4%), with no comorbidities (78/92, 84.8%), and the mean age was 34.1 years (range, 18-72 years). The data collected were not completely reported in all the cases; therefore, the variables were adjusted for each other. The lack of information may be due to cases where the person who injected the filler was not the ophthalmologist who conducted the case and reported the experience.

Hyaluronic acid was the most common filler used (105/215, 48.8%), followed by autologous fat (65/215, 30.2%) and calcium hydroxyapatite (14/215, 6.5%). When the filler was injected in a single region of the face, the preferred site was the nose (65/178, 36.5%), glabella (48/178, 27.0%), forehead (36/178, 20.2%), and nasolabial fold (13/178, 7.3%). The filler was injected using a needle in 13 (56.5%) of 23 cases and a cannula in 10 (43.5%) of 23 cases, with diameter of 0.23-1.00 mm in 22 of 32 patients (68.8%). In 12 of 85 cases, a nonmedical person was responsible for the procedure (14.1%).

All the cases resulted in visual loss. The most common associated symptoms were eye pain (83/216, 38.4%), hea dache (26/216, 12.0%), and nausea/vomiting (26/216, 12.0%). Neurological symptoms were present in 35 (16.2%) of the 216 patients. At initial presentation, 56.4% of the patients also had ptosis and 50.0% also had ophthalmoplegia. The BCVA at the initial visit was <20/200 in 195 (90.3%) of the 216 eyes. Bilateral visual loss was reported in five patients. Table 1 summarizes the ophthalmologic examination results. Slit-lamp examination revealed conjunctiva injection in 20.2%, corneal edema or opacity in 27.5%, anterior chamber inflammation in 12.8%, hypopyon or hyphema in 2.8%, and emboli in conjunctiva vessels in 4.6%.

**Table 1:** Eye examination results of the patients who presented with visual loss after cosmetic facial filler injection

Initial BCVA N=216	n	%
NLP - 20/200 or blindness	195	90.3%
20/160-20/80	0	0.0%
20/63-20/32	11	5.1%
20/25-20/12	10	4.6%
Mean, logMAR		2.57 ± 0.83
**External examination, N=218**	**n**	%
Ptosis	123	56.4%
Ophthalmoplegia	109	50.0%
Pupillary abnormality	94	43.1%
Skin change	98	45.0%
Strabismus	26	11.9%
**Slit-lamp examination, N = 109**	**n**	%
Corneal edema/opacity	30	27.5%
Conjunctiva injection	22	20.2%
Anterior chamber inflammation	14	12.8%
Chemosis	11	10.1%
Subconjunctival hemorrhage	9	8.3%
Iris atrophy	5	4.6%
Emboli visible	5	4.6%
Hypopyon/hyphema	3	2.8%
**Final BCVA, N=209**	**n**	%
NLP - 20/200 or blindness	172	82.3%
20/160-20/80	3	1.4%
20/63-20/32	14	6.7%
20/25-20/12	20	9.6%
Mean, logMAR		2.37±1.08
**Tonometry, N = 35**	**n**	%
1OP <5 mmHg	8	22.9%
1OP 6-10 mmHg	6	17.1%
1OP 11-17 mmHg	6	17.1%
Reported as “normal”	15	42.9%
**Site of occlusion, N = 238**	**n**	%
OAO	77	32.4%
CRAO	54	22.7%
BRAO	18	7.6%
PCAO	22	9.2%
Generalized PCAO	13	5.5%
Localized PCAO	8	3.4%
ION	12	5.0%
Other^1^	14	5.9%
Unknown	41	17.2%

I Anterior chamber injection (1), anterior segment ischemia (1), optic perineuritis (2), third nerve palsy (1), parietal and occipital lobe infarctions (1), lacrimal artery occlusion (1), BRAO and localized PCAO (2), BRAO and posterior ION (1), and other distal occlusions (4).

BCVA= best-corrected visual acuity; BRAO= branch retinal artery occlusion; CRAO= central retinal artery occlusion; ION= ischemic optic neuropathy; NLP= no light perception; OAO= ophthalmic artery occlusion; PCAO= posterior ciliary artery occlusion.

Fundoscopy findings were described in 117 eyes. Seventy-six patients (77 eyes, 32.4%) had OAO, of whom 33 had brain infarction, which represented 73.3% of the brain lesion cases. Filler was injected in the glabella in 18 patients (23.7%), the nose in 15 (19.7%), and the forehead in 12 (15.8%). The filler type was autologous fat in 42 cases (55.3%), hyaluronic acid in 21 (27.6%), and calcium hydroxyapatite in three (3.9%). All the patients had a final visual acuity of <20/200, and 62 eyes (80.5%) had no light perception at final follow-up. Fifty-three patients (54 eyes, 22.7%) were diagnosed as having CRAO. Seventeen patients (32.1%) received autologous fat injections; 13 (24.2%), hyaluronic acid; and two (3.8%), calcium hydroxyapatite. Among the 18 patients (7.6%) who had BRAO, two (11.1%) received autologous fat injection; 10 (55.5%), hyaluronic acid; and one (5.5%), calcium hydroxyapatite. Eight patients (44.4%) had a final visual acuity ≥20/25. Thirteen patients (5.5%) had a generalized PCAO, and their final visual acuity was ≤20/200. Eight patients (3.4%) had a localized PCAO, of whom three had a visual acuity of 20/25 at final presentation and one reported full recover.

Iatrogenic ION may occur owing to distal occlusions of the small vessels that supply the optic nerve. In this review, we found six cases of posterior ION (2.5%) and six cases of anterior ION (2.5%). One patient had a filler injection in the anterior chamber, which was performed by a physician but not an ophthalmologist^(50)^. The patient had a good outcome after irrigation and aspiration of the filler. One patient had presumed occlusion of the lacrimal artery^(64)^. The other causes of visual loss included anterior segment ischemia, optic perineuritis, third nerve palsy, and other distal occlusions.

The location and extent of the artery occlusion are difficult to estimate when angiography was not available. Another proposed classification divides the site of occlusion into diffuse (OAO, generalized PCAO, and CRAO) and localized (BRAO, localized PCAO, ION, and other distal occlusions)^(44)^. Thirty-seven patients (20.1%) had localized occlusions, and 147 (79.9%) had diffuse occlusions.

Optical coherence tomography (OCT) findings were consistent with the respective diagnoses. Twenty-three cases had OCT descriptions, which included macular edema, inner retinal edema, attenuation of all retinal layers, hyper-reflective deposits in the retinal vessels (compatible with emboli), and decreased choroidal thickness. In one case, paracentral acute middle maculopathy was reported^(74)^.

The time to presentation for a second opinion varied widely, from immediate management to >3 weeks. Systemic corticosteroids (33.9%) and hyaluronidase (30.1%) were the most common treatment agents. Hyaluronidase was injected superficially (subcutaneously or in the same site of the filler injection) in 21 patients, intra-arterially in 27, retrobulbarly in 16, and at the supratrochlear/ supraorbital notch in two. Other strategies and final sequelae are described in Table 2. Thirteen eyes evolved to phthisis bulbi (10.7%). Thirteen patients had neurological sequelae (10.7%), which included aphasia, hemiparesia, hemiplegia, weakness, and impaired memory retrieval. One patient died 4 days after the autologous fat injection in the glabella that caused acute infarction of the left cerebral hemisphere^(13)^.

**Table 2:** Management of visual loss due to filler injection and sequelae

Management, N = 186	n	%
Observation	35	18.8%
Steroids	63	33.9%
Hyaluronidase	56	30.1%
Anticoagulant	41	22.0%
1OP lowering agents	38	20.4%
Ocular massage	36	19.4%
Oxygen therapy	27	14.5%
Thrombolysis	26	14.0%
AC paracentesis	19	10.2%
Vasodilator	14	7.5%
Other^1^	3	1.6%
**Sequelae, N = 122**	**n**	%
Neurological sequelae	13	10.7%
Optic disk atrophy	19	15.6%
Fibrous membrane	15	12.3%
Phthisis bulbi	13	10.7%
Strabismus	12	9.8%
Retinal/choroidal atrophy	10	8.2%
Pupillary abnormality	8	6.6%
Visual field defect	7	5.7%
Ophthalmoplegia	6	4.9%
Retinal detachment	3	2.5%
Carotid cavernous fistula	1	0.8%

I Irrigation and aspiration of the filler (1) and decompressive craniectomy (2). AC= anterior chamber; IOP= intraocular pressure.

Overall, most cases had a poor visual prognosis. The mean time of follow-up was 5.1 months. At final presentation, 172 patients (82.3%) had a severe visual impairment (BCVA ≤20/200) in at least one eye. This number can be higher because final vision or changes in visual acuity were not reported or mentioned in 19 eyes. The clinical features associated with worse visual acuity at final presentation were injection of autologous fat (p<0.001), larger diameter of the injection instrument (p=0.040), eye pain (p=0.023), ptosis (p=0.012), neurological symptoms (p=0.005), pale optic disk (p=0.001), and visible emboli in the conjunctiva (p=0.006) at initial evaluation, and brain infarction (p<0.001).

Among the 19 patients who achieved a final visual acuity of 20/20 at final presentation or reported full recovery, seven had BRAO; one, PION; one, localized PCAO; one, anterior chamber injection; one, anterior segment ischemia; two, distal occlusion (probably anterior ciliary); one, third nerve palsy; one, optic perineuritis; and four, no complete eye examination that could have elucidated the diagnosis. Four patients already had good visual acuity at presentation but complained of visual field defects. Of the patients who received filler injections, 13 (68.4%) received hyaluronic acid; three (15.8%), calcium hydroxyapatite; one (5.3%), botulinum toxin A; and two, unknown filler. None of them had a brain infarction. Management included hyaluronidase in eight (of 13 patients, 61.5%), corticosteroids in seven (36.8%), oxygen therapy in four (21.0%), and observation in two (10.5%). Hyaluronidase was injected subcutaneously in three patients, retrobulbarly in one, at the supratrochlear/supraorbital notch in two, and subcutaneously and retrobulbarly in two. Three patients underwent hyaluronidase treatment immediately after the visual loss and reported relief of symptoms after the enzyme injection^(56,75,78)^.

When comparing the two most common type of filler, we found that the patients who received autologous fat injections tended to be older, possibly because the procedure can be combined with other aesthetic procedures such as liposuction, which is common in this age group. The diameter of the cannula or needle was larger when fat was injected. Autologous fat was also related to diffuse occlusions, mainly OAO, brain infarction, worse visual acuity at presentation, worse visual outcomes, lower visual gaine, and neurological sequelae. The patients who received hyaluronic acid injection tended to have skin changes and ptosis at clinical presentation (Table 3).

**Table 3:** Comparative table of clinical characteristics of filler-induced visual loss by autologous fat and hyaluronic acid

	Autologous fat	Hyaluronic acid	p Value
Age, years	35.7 ± 12.1	29.8 ± 8.0	p=0.002
Diameter of cannula/needle	1.18 ± 0.62	0.43 ± 0.11	p<0.001
Occlusion type			
Diffuse occlusion	61/64 (95.3%)	44/68 (64.7%)	p<0.001
Localized occlusion	3/64 (4.7%)	24/68 (35.3%)	
OAO	42/64 (65.6%)	21/68 (30.9%)	p<0.001
Generalized PCAO	2/64 (3.1%)	7/68 (10.3%)	
CRAO	17/64 (26.6%)	13/68 (19.1%)	
Localized PCAO	0	4/68 (5.9%)	
BRAO	2/64 (3.1%)	10/68 (14.7%)	
ION	1/64(1.6%)	7/68 (10.3%)	
Initial presentation			
Ptosis	24/62 (38.7%)	64/99 (64.6%)	p=0.001
Ophthalmoplegia	32/62 (51.6%)	58/99 (58.6%)	p=0.386
Strabismus	5/62 (8.1%)	11/99 (11.1%)	p=0.529
Pupillary abnormality	23/62 (37.1%)	49/99 (49.5%)	p=0.124
Skin change	13/62 (21.0%)	49/99 (49.5%)	p<0.001
Anterior segment ischemia	5/34 (14.7%)	15/42 (35.7%)	p=0.062
Brain infarction	29/65 (44.6%)	11/105 (10.5%)	p<0.001
Initial BCVA (logMAR)	2.84 ± 0.57	2.48 ± 0.89	p<0.001
NLP - 20/200	55/57 (96.5%)	89/98 (90.8%)	
Final BCVA (logMAR)	2.83 ± 0.64	2.16 ± 1.17	p<0.001
NLP - 20/200	54/57 (94.7%)	74/94 (78.7%)	
Visual gain (logMAR)	-0.01 ± 0.85	-0.30 ± 0.84	p=0.001
Sequelae			
Neurological	8/33 (24.2%)	4/59 (6.8%)	p=0.024
Phthisis bulbi	2/33 (6.1%)	11/59(18.6%)	p=0.125

Data was compared by Mann-Whitney U test for continuous variables and by chi-square test or Fisher’s exact test for categorical values.

BCVA= best-corrected visual acuity; BRAO= branch retinal artery occlusion; CRAO= central retinal artery occlusion; ION= ischemic optic neuropathy; NLP= no light perception; OAO= ophthalmic artery occlusion, PCAO: posterior ciliary artery occlusion.

The profiles of the patients changed over time (Table 4). When compared with the first case published until 2014, the cases reported in the period 2015-2020 were comprised of younger patients (mean age, 37.6 years vs 31.1 years), more females, more frequent use of hyaluronic acid, and more frequent injections by unlicensed professionals. The preferred site of injection changed from the glabella to the nose and forehead. Among the patients in the latter period, the prevalence rates of brain infarction, use of the observational approach, and ophthalmic artery occlusion were lower, probably because of the less frequent use of autologous fat.

**Table 4:** Comparison of the profiles of patients with facial filler-induced visual loss between periods

	1988-2014,n (%)	2015-2020n (%)	p Value
Age (years)	37.6 (18-72)	31.1 (20-65)	p<0.001
Female	93 (89.4)	117(96.7)	p=0.034
Male	11 (10.6)	4 (3.3)	
Filler type			p<0.001
Hyaluronic acid	28 (26.9)	77 (74.0)	
Autologous fat	52 (50.0)	13(12.5)	
Calcium hydroxyapatite	6 (5.8)	8 (7.7)	
Person who performed the procedure			p=0.006
Physician	58 (92.1)	15 (68.2)	
Nonmedical injector	5 (7.9)	7(31.8)	
Site of injection			
Glabella	35 (34.0)	14(11.5)	p<0.001
Nose	21 (20.4)	43 (35.2)	p=0.014
Forehead	7 (6.8)	29 (23.8)	p=0.001
Nasolabial fold	6 (5.8)	5(4.1)	p=0.550
Diagnosis			
OAO	49 (46.7)	28 (32.2)	p<0.001
CRAO	25 (23.8)	29 (33.0)	
BRAO	13 (12.4)	5 (5.7)	
PCAO	12 (11.4)	10(11.5)	
ION	5 (4.8)	7 (8.0)	
Brain infarction	28 (25.9)	17(13.6)	p=0.017
Initial BCVA (logMAR)	2.41 ± 0.97	2.71 ± 0.64	p=0.082
Final BCVA (logMAR)	2.31 ± 1.13	2.42 ± 1.04	p=0.994
Visual gain (logMAR)	-0.11 ± 0.81	-0.32 ± 0.92	p=0.057
Treatment			
Observation	28 (27.8)	7 (8.3)	p=0.001
Steroids	31 (30.4)	32 (38.1)	p=0.269
Hyaluronidase	3 (2.9)	53 (63.1)	p<0.001

Data were compared using the Mann-Whitney *U* test for continuous variables and by the chi-square test or Fisher exact test for categorical values.

BCVA=: best-corrected visual acuity; BRAO= branch retinal artery occlusion; CRAO= central retinal artery occlusion; ION= ischemic optic neuropathy; IOP= intraocular pressure; NLP= no light perception; OAO= ophthalmic artery occlusion; PCAO= posterior ciliary artery occlusion.

## DISCUSSION

An exponential increase in the number of cases of blindness after essentially cosmetic procedures has been published in the literature, mainly affecting young people of working age. Most studies that described this complication were conducted in Asian countries, consistent with the high increase in article output from Asia. PubMed was the selected database because of its highly comprehensive features and high popularity as an online database resource among healthcare professionals.

### Mechanisms and anatomical consideration

Filler-induced visual loss is usually related to occlusion of arteries from the ophthalmic artery system, which occurs owing to inadvertent intravascular injection in small branches and retrograde embolism. If the injector applies a pressure higher than the arterial pressure of the patient, the filler will flow through the artery. When the embolus is released, the filler will propagate toward the distal branches, occluding it. The typical injection pressures applied by experienced injectors were significantly lower than that required to cause propagation of the filler and the mean arterial pressure in a cadaver study^(101)^. Increased intraocular pressure can also block arterial blood flow and produce the same clinical course. Coagulation of the filler material can worsen the occlusion.

**Figure 2:**
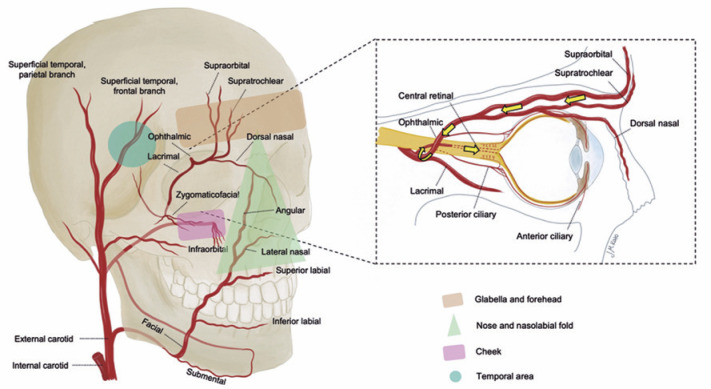
Areas of the face most commonly related to filler-induced blindness and the respective arteries affected. The arrows indicate the filler flow through the arteries and retrograde embolism until it reaches the central retinal artery.

The glabella and forehead (supratrochlear and supraorbital arteries), nose and nasolabial fold (dorsal nasal, lateral nasal, angular, and facial arteries), temple area (superficial temporal artery and middle temporal vein), and middle cheek (zygomaticofacial and infraorbital arteries) and the respective arteries affected are examples of areas at risk^(102,103)^. Figure 2 illustrates the most common ophthalmic branches damaged by fillers and its anatomical course. The common areas of occlusion could also be related to the fact that most studies were conducted in Asians and cultural disparity causes differences in cosmetics goals. Asian people seek an oval facial shape, preferring augmentation of the midline features (forehead, glabella, nose, medial cheeks, and chin), which include areas at high risk of vascular occlusion^(104)^. These areas may also present a large variety of branching patterns^(105)^, and previous trauma or surgery can lead to unpredictable vascular anastomosis. Therefore, every area in the face is susceptible to ophthalmic occlusion^(105)^, and even experienced injectors may face this complication.

Particle size may explain the difference in occlusion site depending on the filler type. Hyaluronic acid particles range from 400 to 750 µm in size^(106)^ and are more likely to occlude smaller arteries. Only 50 µL of hyaluronic acid can occlude small vessels, which can be superficial, occurring at a depth of 1.5 mm from the skin surface^(107)^. Fat particles are larger and may block larger vessels such as the ophthalmic artery. A cadaver study showed that the diameter of the ophthalmic artery was approximately 2 mm, and the diameters of the supratrochlear, supraorbital, dorsal nasal, and angular arteries were approximately 1 mm^(102)^. The lacrimal artery is one of the largest branches of the ophthalmic artery, and only one case of presumed occlusion occurred in the lacrimal branch. Moreover, autologous fat injection was associated with the use of larger diameter cannula/ needles possibly because of the larger particles in autologous fat.

### Prevention

According to major reviews, the key strategies to avoid visual loss from fillers are as follows:

1. Prefer local anesthesia, as general anesthesia may delay the onset of patient complaints.
2. Consider using local epinephrine for its vasoconstriction properties.
3. Prefer blunt cannulas to avoid arterial puncture and small syringes to prevent injection of a large amount at a time.
4. Apply directly on bone or superficially in the dermis, as the subcutaneous plane is where the vasculature is commonly located. Avoid the most common depth patterns of the vessels at risk.
5. Aspirate before injecting, and inject very slowly with minimal pressure.
6. Apply digital pressure with the nondominant hand to occlude the artery at risk during the injection. Release the pressure after all the filler has been injected.
7. Use a small volume at a time, and try to move the needle/cannula to prevent depositing a large quantity of filler in a single location.

Some strategies, however, are limited owing to the usual presentation of the commercially available hyaluronic acid. The use of local epinephrine, for example, requires manipulation of the filler content, but the filler is packaged in a ready-to-go syringe, and the package includes needles, not cannulas.

### Management

Treatment aims at restoration of eye perfusion within 90 minutes, as after this period, the damage to the retina becomes irreversible^(108)^. A recent study reported that even 12-15 minutes may be fatal owing to damage to the retinal ganglion cells^(109)^.

First, the procedure has to be immediately stopped if the patient complains of pain or visual change. Injectors should promptly recognize symptoms and signs of vascular occlusion. Ocular massage and warm compression can be initial strategies to be taken during transfer for an ophthalmologic evaluation^(110)^. Brain imaging may be necessary to rule out brain infarction. An ophthalmologic examination should be performed to confirm the diagnosis, including pupil examination, extraocular movements, slit-lamp examination, and fundoscopy. Recording of visual acuity at initial presentation and the presumed site of occlusion is important. Some case reports informed on vision recovery but did not perform any objective measurement of visual acuity.

To date, no evidence-based strategy has been established to deal with visual loss after facial filler injection. Case reports with good visual outcomes are usually related to minor, localized occlusions. The following are the strategies described in previous reports:

1. **Intraocular pressure reduction**, to increase blood flow, dislodge the embolus peripherally and restore perfusion. Ocular massage, topical agents (beta-blockers, carbonic anhydrase inhibitors, and prostaglandin analogs), oral acetazolamide, intravenous mannitol, and anterior chamber paracentesis are examples. Ocular massage should be performed for 10-15 seconds, followed by a sudden release. A recent consensus among calcium hydroxyapatite experts advised that if a large bolus of >0.1 mL was injected, ocular massage should not be performed until vasodilation measures have been administered because it would increase the area of embolization^(111)^. Anterior chamber paracentesis is usually performed with caution not to touch the lens, using a 28- to 30-gage needle in the 9-10 o’clock direction in the right eye, parallel to the iris;
2. **Vasodilation** (warm compression, carbon dioxide, sublingual nitroglycerin, intravenous alprostadil, prostaglandin E1, and pentoxifylline), also to increase blood flow;
3. **Hyperbaric oxygen**, to increase oxygen delivery;
4. **Anticoagulant** (aspirin, pentoxifylline), to avoid blood clotting upstream to the filler embolus;
5. **Steroids** (intravenous dexamethasone and oral prednisone), to decrease inflammation;
6. **Thrombolysis** (intravenous or intra-arterial). A meta-analysis study suggested beneficial outcomes of intravenous fibrinolysis for central retinal artery occlusion not induced by filler injection when used within 4.5 hours of symptom onset^(112)^. Embolic materials such as dermal fillers, however, seem to be resistant to this therapy;
7. **Hyaluronidase**, to dissolve hyaluronic acid. It can be applied subcutaneously at a high dose, at the site of injection and surrounding areas. The enzyme can diffuse across the arterial wall, degrading the hyaluronic acid without the need to directly cannulate the artery. Hyaluronidase can also be used at the retrobulbar space to get closer to the area of occlusion. In nine cases, immediate injection was performed in the same area where the filler was injected, and then a retrobulbar injection was performed when the patient reached the ophthalmology department. Two patients recovered their vision after retrobulbar injection of hyaluronidase^(75,96)^. Retrobulbar injection is performed in the inferior-lateral orbital rim, penetrating along the orbital floor, aiming superiorly to target the intraconal space. Approximately 2-4 mL should be injected. Volume should not increase the intraocular pressure, as it could force the embolus further into the arteries. Another technique targets the area of the supraorbital or supratrochlear notch to cannulate the arteries and push hyaluronidase retrogradely. Two cases showed vision recovery using this technique^(56,78)^.

To provide a prompt treatment, a well-established flowchart for managing complications is mandatory. Easily accessible professionals and services must also be available for the patient with suspected arterial obstruction.

Ptosis, ophthalmoplegia, and strabismus usually resolves during follow-up, as muscle and nerves can regenerate. Strabismus surgery may be necessary in cases with sensory strabismus^(113)^. Appropriate ophthalmologic follow-up is essential owing to the risk of neovascularization. Good physician-patient relationship is also crucial to manage possible psychiatric demands of the patient.

### Trends in visual loss induced by filler injection

Filler-induced visual loss in the last 5 years was associated with younger people (p<0.001) and female sex (p=0.034). Even though more men are seeking cosmetic procedures, accounting for 9.3% of hyaluronic acid injections in 2018^(1)^, a higher percentage of women had vision complications (96.7%). This may be because injecting fillers in the central face tends to be more feminizing, and these areas involve the most common sites related to ophthalmologic complications.

The main site of complications continues to be in the central face but no longer in the glabella, probably owing to the many previous reports identifying this area as the most dangerous. In addition, as younger people are seeking cosmetic procedures, they may tend to receive botulinum toxin injection in the glabella for preventing deep wrinkles.

A trend study reported that a high number of people are seeking cosmetic procedures with hyaluronic acid instead of fat, a finding similar to those of previous reviews^(114)^. Many complications related to the filler are expected. Hyaluronic acid filler was approved by the Food and Drug Administration in the early 2000, and since then, it has revolutionized the filler market owing to its long-lasting and low immunogenicity characteristics. It allows the use of smaller-diameter cannulas/needles; therefore, in cases of vascular occlusions, it is related to only localize damage and better visual prognosis. However, the ease of access to hyaluronic acid allowed a wide range of professionals to perform filler injection without the appropriate care, increasing the incidence rate of complications. Fat, on the other hand, is usually applied by surgeons, who should be more aware of its proper use to prevent severe complications. The later period also had a higher percentage of unlicensed professionals responsible for cases of filler-induced blindness (8.3% compared with 30.4%). This points out to an increased awareness of the danger caused by unauthorized practitioners.

Fortunately, the later period had lower incidence rates of brain infarction and OAO. We observed a more proactive approach in the later period, mainly represented by the use of hyaluronidase and explained by the wider variety of treatment options. However, evidence-ba sed strategies are still lacking, and most patients have a poor prognosis. Despite the exponential increase of notifications since 2012, the high incidence of blindness following injection has been sub-notified.

This review summarizes the profiles of 233 patients with visual loss induced by aesthetic filler injection and allows a detailed evaluation of autologous fat and hyaluronic acid injections. When analyzing the changing trends over the years, we found that the continuous de velopment of fillers has provided a wide variety of available preventive and treatment methods. However, though rare, blindness as a complication of a cosmetic procedure is unacceptable, and further evidence-based studies and strategies are necessary to reduce its incidence and consequences.
